# A disulfidptosis-related lncRNA prognostic model to predict survival and response to immunotherapy in lung adenocarcinoma

**DOI:** 10.3389/fphar.2023.1254119

**Published:** 2023-09-25

**Authors:** Hai-Bo Zhang, Jian-Yan Pan, Tao Zhu

**Affiliations:** ^1^ Department of Pharmacy, Hangzhou Women’s Hospital, Hangzhou Maternity and Child Health Care Hospital, Hangzhou, China; ^2^ Department of Birth Health and Genetics, The Reproductive Hospital of Guangxi Zhuang Autonomous Region, Nanning, China; ^3^ Department of Pharmacy, The Second Affiliated Hospital, Zhejiang University School of Medicine, Hangzhou, China; ^4^ Key Laboratory of Intelligent Pharmacy and Individualized Therapy of Huzhou, Department of Pharmacy, Changxing People’s Hospital, Hangzhou, China

**Keywords:** lung adenocarcinoma, disulfidptosis, lncRNA, prognosis, immunotherapy

## Abstract

**Background:** Lung adenocarcinoma (LUAD) is the major subtype of lung cancer and has a poor prognosis. Disulfidptosis is a novel regulated cell death form characterized by aberrant disulfide stress and actin network collapse. This study aimed to identify disulfidptosis-related lncRNAs, and predict LUAD patients’ prognosis and response to antitumor therapies by establishing a disulfidptosis-related lncRNA model.

**Methods:** Transcriptome and clinical data of LUAD patients were obtained from the TCGA database. Pearson correlation and Cox regression analysis was used to identify disulfidptosis-related lncRNAs associated with overall survival. LASSO regression analysis was adopted to construct the prognostic model. GO, KEGG and GSEA analysis was used to identify cellular pathways related to this model. Immune cell infiltration was investigated by ESTIMATE and CIBERSORT algorithms. Tumor mutational burden (TMB) and its association with model-derived risk score were analyzed using simple nucleotide variation data. Patients’ response to immunotherapy and other antineoplastic drugs was predicted by the TIDE algorithm and GDSC tool, respectively.

**Results:** We identified 127 disulfidptosis-related lncRNAs, and a prognostic model that consists eight of them (KTN1-AS1, AL365181.3, MANCR, LINC01352, AC090559.1, AC093673.1, AP001094.3, and MHENCR) was established and verified. The prognostic model could stratify LUAD patients into two distinct risk-score groups. A high risk score was an independent prognosis factor indicating poor overall survival, and correlated with reduced immune cell infiltration, high TMB, and lower activity of tumor immune response. Immune checkpoint blockade might bring more survival benefits to the high-risk LUAD patients, whereas low-risk patients might be more responsive to targeted therapy and diverse kinase inhibitors.

**Conclusion:** We established a disulfidptosis-related lncRNA model that can be exploited to predict the prognosis, tumor mutational burden, immune cell infiltration landscape, and response to immunotherapy and targeted therapy in LUAD patients.

## Introduction

Lung cancer remains one of the leading causes of global cancer incidence and mortality (Sung et al., 2021; Zheng et al., 2023). Adenocarcinoma is the main histological type of non-small cell lung cancer (NSCLC) and accounts for around 40% of all lung cancers (Duma et al., 2019). Early detection and diagnosis are directly related to clinical outcome, and its failure often leads to miss of the optimal opportunity of clinical intervention. For patients with stage I or II disease, surgical resection is recommended. For patients at advanced stages, besides traditional radiotherapy and chemotherapy, systemic therapeutic strategies comprising targeted therapy and immunotherapy are optional for NSCLC treatment according to the gene mutation scenarios (e.g., *EGFR* mutation, *ALK* translocation) and expression of programmed cell death protein-ligand 1 (PD-L1) (Duma et al., 2019). Lung adenocarcinoma (LUAD) is molecularly and phenotypically diverse, and approximately 60% of LUAD have an oncogenic driver mutation that in many cases is associated with certain clinicopathologic features and predicts treatment response (Sholl, 2015; Tavernari et al., 2021). For example, *KRAS* and *KEAP1* are among the most frequently mutated genes in LUAD. *KEAP1* mutations confers shorter overall survival (OS) in *KARS* mutant LUADs in response to anti-PD-(L)1 immunotherapy (median OS (95%CI): 4.8 months (4.0–8.0) for *KEAP1* mutant versus 18.4 months (14.9–221.7) for *KEAP1* wild-type), but not in *KRAS* wild-type LUADs (Ricciuti et al., 2022). In another LUAD cohort treated with immune checkpoint inhibitors, *KEAP1* inactivation mutations due to somatic mutation and loss of heterozygosity are correlated with worse clinical outcomes and an immune-excluded phenotype (Scalera et al., 2023). The survival rate remains dismal despite of advances in genotype-based diagnosis and therapy modalities (Zhang et al., 2019). To improve LUAD management, a solid understanding of molecular events that correlate with LUAD malignant degree is necessary.

Resisting regulated cell death is a hallmark of cancer (Hanahan, 2022). Increasing evidence shows that different regulated cell death forms can affect cancer progression and response to therapy (Peng et al., 2022). For example, ferroptosis, characterized by iron-dependent lipid hydroperoxide accumulation, was found to be implicated in T cell immunity and contribute to immunotherapy efficacy (Wang. et al., 2019a). Disulfidptosis is a recently identified regulated cell death type induced by aberrant accumulation of intracellular disulfides in SLC7A11-overexpressing cells under a glucose starvation condition (Liu et al., 2023). Increased SLC7A11-mediated cystine uptake, in couple with glucose starvation, causes severe disulfide stress and facilitates aberrant disulfide bonding in actin cytoskeleton proteins, leading to actin filament contraction and detachment from the plasma membrane (Liu et al., 2023). A recent study by Chen et al. highlights that disulfidptosis plays a role in regulating bladder cancer progression and therapy efficacy (Chen et al., 2023). However, it remains unclear whether disulfidptosis is involved in LUAD progression and affects prognosis of LUAD patients.

Long non-coding RNAs (lncRNAs) are transcripts of more than 200 nucleotides that are not translated into proteins. LncRNA-encoding loci are among the most numerous regulatory and functional units in the non-coding regions of the genome (Uszczynska-Ratajczak et al., 2018). They play critical roles in regulating gene expression and protein function by interacting with DNA, RNA and proteins (Liu et al., 2015; Blank-Giwojna et al., 2019; Statello et al., 2021). The involvement of lncRNAs in gene expression regulation under pathological conditions suggests that they are related to a broad range of diseases. In terms of LUAD, a growing number of studies have demonstrated that lncRNAs promote disease progression (e.g., UPLA1 and LINC00628) and immune evasion (e.g., SChLAP1), and can serve as prognosis biomarkers and potential drug targets (Xu et al., 2019; Han et al., 2020; Du et al., 2021).

In this study, we aimed to identify disulfidptosis-related lncRNAs that affect prognosis of LUAD patients. We constructed and validated a prognostic model based on disulfidptosis-related lncRNAs, and this model exhibits high accuracy in predicting survival rate (area under the curve (AUC) for 1 year survival: 0.703). Moreover, the model-derived risk score can be used to evaluate tumor immune micro-environment landscape and sensitivity to immunotherapy and chemotherapy. Besides tumor stage, our prognostic model is an independent factor with potential to identify patients with high risk (hazard ratio (HR): 1.245, 95% CI: 1.167–1.328, *p* < 0.001). Our findings demonstrate key regulatory roles of disulfidptosis-related lncRNAs in LUAD progression and provide potential targets for precision treatment of LUAD.

## Materials and methods

### Data acquisition

The RNA-sequencing (RNA-seq)-based transcriptome profiling data, clinical information and somatic mutation data of over 500 LUAD patients were downloaded from The Cancer Genome Atlas (TCGA) database. Normal control samples were excluded for further analysis. LUAD cases with insufficient information about survival time, age, and tumor stage were also removed.

### Screening for disulfidptosis-related lncRNAs

We obtained 25 disulfidptosis-related genes based on previous studies (Liu et al., 2023; Yang et al., 2023; Zhao et al., 2023). Pearson correlation analysis was performed to identify lncRNAs that exhibit co-expression patterns with the disulfidptosis-related genes, with the absolute value of correlation coefficient >0.4 and *p* < 0.001 as the screening threshold. These lncRNAs were defined as disulfidptosis-related lncRNAs.

### Establishment and validation of a disulfidptosis-related lncRNA prognosis model

A total of 507 LUAD samples with survival information were randomly divided into two groups, one for model construction (the training group, n = 254) and one for model validation (the test group, n = 253). In the training group, disulfidptosis-related lncRNAs that were associated with patients’ overall survival were obtained by performing univariate Cox regression analysis. After LASSO regression analysis to determine lncRNAs with minimum deviation, a prognostic model based on eight disulfidptosis-related lncRNAs was established through multivariate Cox regression analysis. The risk score was the sum of products of the expression value of each of the eight lncRNAs and its regression coefficient, $\text{risk score}=\sum_{i=0}^{n}\beta i\mathrm{Exp}i$ (Zhu et al., 2020). Based on the median risk score, patients were grouped into high- and low-risk subgroups, and survival analysis was carried out to evaluate the significance of the prognostic model. Samples in the test group were used to validate the reliability of this prognostic model. Multivariate Cox regression analysis was conducted to evaluate whether the risk score derived from the model is an independent prognostic factor of LUAD patients.

### Functional enrichment analysis of differentially expressed genes

Differentially expressed genes (DEGs) were screened between the high and low risk groups, according to the screening criteria: |log2 fold change| > 1 and false discovery rate (FDR) < 0.05. After that, Gene Ontology (GO) and Kyoto Encyclopedia of Genes and Genomes (KEGG) pathway analyses were carried out to gain insights into possible molecular events that distinguish between high- and low-risk groups. GO terms or KEGG pathways were considered significantly enriched when FDR was less than 0.05. With a focus on Gene Ontology gene sets, gene set enrichment analysis (GSEA) was also performed based on gene expression profiles between the two groups. A gene set was considered enriched when *p*-value and FDR were less than 0.05 and 0.25, respectively.

### Tumor infiltrating immune cells analysis

The ESTIMATE R package was used to analyze the abundances of infiltrating stromal and immune cells in LUAD tissues using gene expression data (Yoshihara et al., 2013). The ESTIMATE algorithm generates three scores based on single sample GSEA, including stromal, immune and estimate scores. Their differences between high and low risk LUAD groups were compared.

The CIBERSORT tool (Chen et al., 2018) was further employed to estimate the abundances of 22 immune cell types in each of the LUAD samples. In addition, single sample GSEA was performed using immune-related gene sets to evaluate multiple immune functions of each sample, and the activities of these immune functions were compared between two risk groups.

### Tumor mutational burden analysis

According to the total number of somatic base substitutions, the tumor mutational burden (TMB) and mutation frequencies in each sample were calculated. Differences in TMB between the high- and low-risk groups of patients were analyzed. According to the median TMB score, LUAD patients were divided into two groups and survival analysis was performed to explore the influence of TMB on patients’ overall survival. The combined effect of TMB and risk score on patient prognosis was also investigated.

### Immunotherapy response and drug sensitivity prediction

We exploited the Tumor Immune Dysfunction and Exclusion (TIDE) platform (Jiang et al., 2018) to predict LUAD patient response to anti-PD-1 and anti-CTLA4 immunotherapy. TIDE prediction scores are negatively associated with immunotherapy response. Differences in response to immunotherapy between the high- and low-risk groups of patients were analyzed by comparing the TIDE scores.

The oncoPredict R package was used for predicting drug sensitivity in LUAD patients based on gene expression data. The required training sets were derived from the Genomics of Drug Sensitivity in Cancer database (GDSC) and downloaded from oncoPredict’s Open Science Framework (https://osf.io/c6tfx/) (Maeser et al., 2021). We used the calcPhenotype function to obtain drug sensitivity scores of each patient. Differences in response to multiple drugs between the high- and low-risk groups were compared based on the drug sensitivity scores.

### Statistical analysis

Data analysis was performed in R (4.2.2). Two-tailed Student’s t*-*test was used to compare statistical differences between two groups. The Kaplan-Meier estimate and log-rank test were used for survival analysis. Unless otherwise indicated, differences were considered statistically significant when *p* < 0.05.

## Results

### Identification of disulfidptosis-related lncRNAs

**FIGURE 1:**
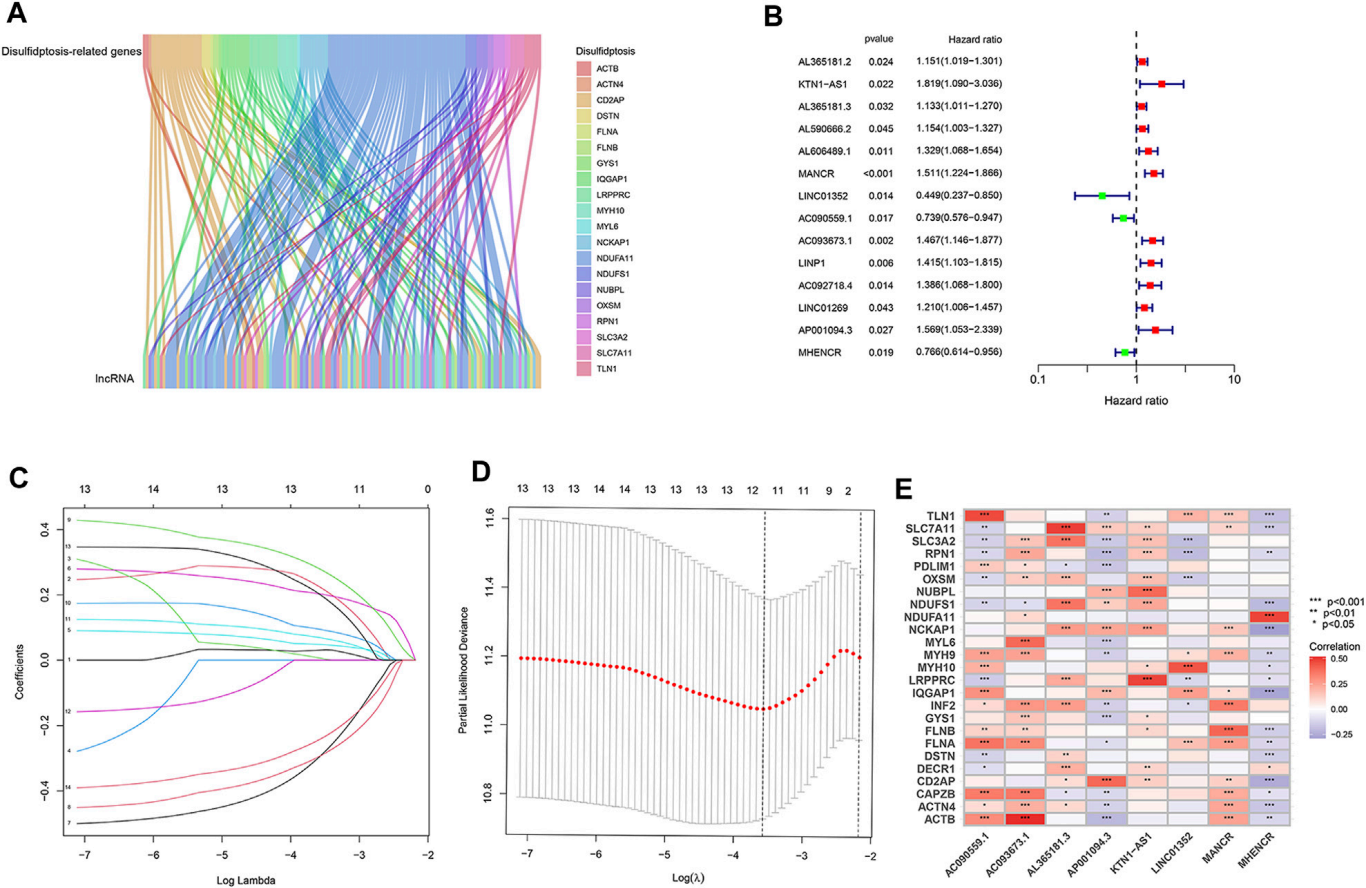
Identification of disulfidptosis-related lncRNAs and construction of prognostic model in LUAD. **(A)** The Sankey diagram showing significant expression correlations of disulfidptosis-related genes with 127 lncRNAs. **(B)** Disulfidptosis-related lncRNAs that affect the overall survival of LUAD patients according to univariate Cox regression analysis. **(C)** LASSO coefficients of the 14 lncRNAs that correlate with overall survival. **(D)** Cross-validation of LASSO regression, the dashed lines denote the optimal log(λ) value. **(E)** Heatmap showing the expression correlation between the eight lncRNAs used for model construction and disulfidptosis-related genes. *, *p* < 0.05; **, *p* < 0.01; ***, *p* < 0.001.

RNA-seq data of LUAD patients were downloaded from TCGA. According to the annotation of gene type, protein coding mRNAs and lncRNAs were distinguished. To identify lncRNAs implicated in disulfidptosis, Pearson correlation analysis was conducted based on expression levels of lncRNAs and 25 disulfidptosis-related genes. Following stringent screening criteria (|Pearson R| > 0.4 and *p* < 0.001), 127 lncRNAs were screened out and their expression were correlated with 20 of the 25 disulfidptosis-related genes (Figure 1A; Supplementary Table S1).

### Establishment of the disulfidptosis-related lncRNA prognostic model

**FIGURE 2:**
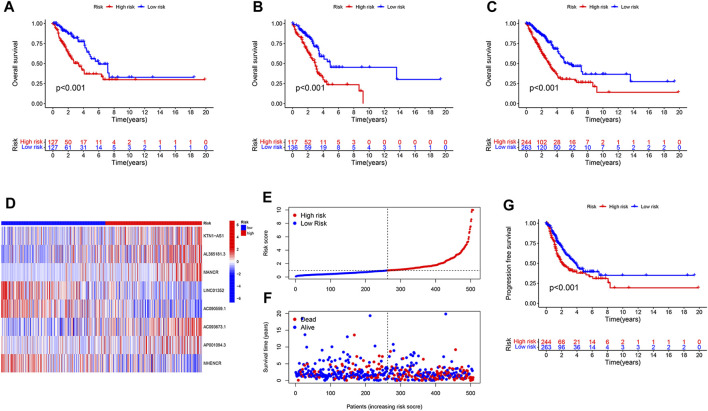
Evaluation and verification of the prognostic value of the disulfidptosis-related lncRNA model. **(A,B)** Kaplan-Meier curves showing the difference in overall survival between high- and low-risk LUAD patients in the training group **(A)** and in the test group **(B)**. **(C,D)** Kaplan-Meier curves showing the difference in overall survival **(C)** and progression free survival **(D)** in the combined LUAD cohort with high-risk and low-risk. **(E)** The risk score of each LUAD patient ordered from low to high is shown. **(F)** Survival status of LUAD patients that are ordered from low to high according to the risk score. **(G)** Heatmap showing expression of the eight disulfidptosis-related lncRNAs in LUAD patients with high-risk or low-risk.

We randomly divided 507 LUAD patients with survival information into two groups, the training group was for model construction and the test group for model validation. Among the 127 lncRNAs, only those associated with LUAD patient survival were considered to be enrolled in model construction. We performed univariate Cox regression analysis to exclude lncRNAs that showed no significant effect on survival, and 14 disulfidptosis-related lncRNAs were left. Of them, three were prognostically favorable lncRNAs and eleven were prognostically unfavorable ones (Figure 1B). Based on these 14 prognosis-associated disulfidptosis-related lncRNAs and using LASSO Cox regression analysis, a prognostic model comprised of 8 disulfidptosis-related lncRNAs was further established (Figures 1C, D). Then we assigned each patient a risk score per the formula of the prognostic model: risk score = (0.433 * KTN1-AS1 expression value (EV)) + (0.099 * AL365181.3 EV) + (0.274 * MANCR EV)—(0.604 * LINC01352 EV)—(0.404 * AC090559.1 EV) + (0.425 * AC093673.1 EV) + (0.374 * AP001094.3 EV)—(0.340 * MHENCR EV). The expression correlations between the 8 lncRNAs and 25 disulfidptosis-related genes were shown in Figure 1E, AC093673.1 and AL365181.3 showed positive associations while AP001094.3 and MHENCR showed negative correlations with most disulfidptosis-related genes. According to the median risk score, the training group was divided into high-risk and low-risk groups. As expected, patients in the high-risk group had shorter overall survival time, demonstrating the prognostic significance of the 8 lncRNAs-based model (Figure 2A). Similar survival analysis results were observed in the test group and after combination of the two groups (Figures 2B, C), which indicates that our prognostic model is reliable. Moreover, the 8 disulfidptosis-related lncRNAs exhibited consistent expression patterns per the risk scores between the training and test groups (Figure 2D).

We merged the training and test groups into one and divided it into two groups. Each patient’s risk score and survival state were shown in Figures 2E, F a high risk score was positively correlated with an increased probability of death. In addition to predicting dismal overall survival, a high risk score also indicated poor progression-free survival (Figure 2G).

### The disulfidptosis-related lncRNA model is an independent prognostic indicator

**FIGURE 3:**
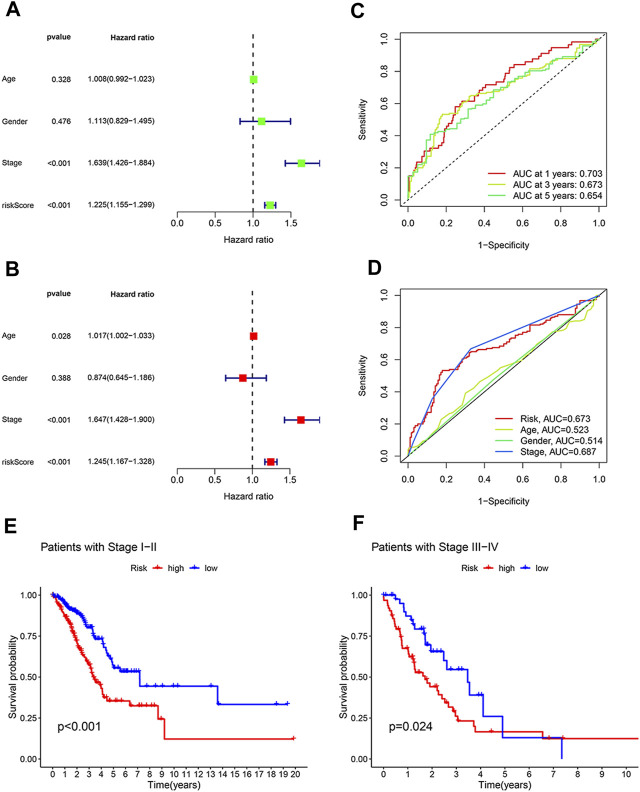
The model based on eight disulfidptosis-related lncRNAs is an independent prognosis indicator with high accuracy. **(A)** Forest plot showing prognostic value of age, gender, tumor stage and the model-derived risk score according to univariate regression analysis. **(B)** Forest plot showing tumor stage and our model-derive risk score are independent prognostic factors based on multivariate regression analysis. **(C)** The prognostic accuracy of our model-derived risk score for predicting 1-year, 3-year, and 5-year survival. **(D)** The accuracy of risk score, tumor stage, age and gender in predicting LUAD patients’ survival. **(E,F)** Kaplan-Meier curves showing the difference in overall survival between high- and low-risk LUAD patients at early stages **(E)** and at advanced stages **(F)**.

We next asked whether our prognostic model was interfered by other clinical factors. We enrolled four clinical features of LUAD patients, including age, gender, tumor stage and risk score, for Cox regression analysis. According to univariate analysis, tumor stage (HR: 1.639, 95% CI:1.426–1.884, *p* < 0.001) and the 8 disulfidptosis-related lncRNAs-based risk score (HR: 1.225, 95% CI: 1.155–1.299, *p* < 0.001) are two hazardous factors that affect prognosis (Figure 3A). Moreover, the multivariate Cox regression analysis suggested that risk score (HR: 1.245, 95% CI: 1.167–1.328, *p* < 0.001), together with tumor stage (HR: 1.647, 95% CI: 1.428–1.900, *p* < 0.001), are independent prognostic factors (Figure 3B). To further evaluate the predictive accuracy of the lncRNA-based prognostic model, ROC curve analysis was performed. The AUC values for 1-, 3-, and 5-year survival are 0.703, 0.673, and 0.654, respectively, indicating high accuracy of our prognostic model (Figure 3C). Of note, our prognostic model is almost as accurate as tumor stage in prognosis prediction, as reflected by similar AUC values (0.673 versus 0.687) (Figure 3D). These results suggest that our disulfidptosis-related lncRNAs-based model can serve as an independent and accurate prognostic indictor.

The practicability of the lncRNA-based model in prognosis prediction of LUAD patients with the same disease stage.

Since both our prognostic model and tumor stage are good survival predictors, we wondered what are the advantages of our model as compared with tumor stage in terms of prognosis prediction. To that end, LUAD patients were divided into early stage group (stage I or II) and advanced stage group (stage III or IV) according to the disease stage. It was found that in both groups, LUAD patients with high risk scores had a poorer overall survival rate than patients with low risk scores (Figures 3E, F). These results suggest that our prognostic model can distinguish between patients at high and low risk, even at the same disease stage.

### Involvement of the disulfidptosis-related lncRNA model in immune regulation

**FIGURE 4:**
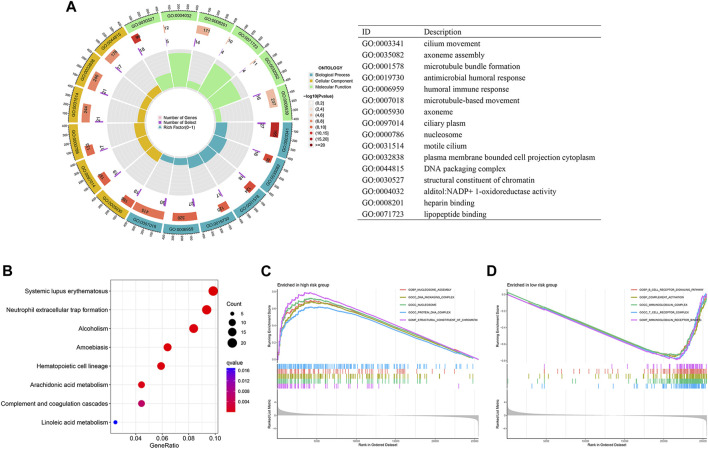
Differential function enrichment between the high- and low-risk LUAD patients. **(A,B)** GO enrichment **(A)** and KEGG **(B)** analysis of differentially expressed genes in the two LUAD groups. **(C,D)** GSEA results showing cellular processes significantly enriched in high-risk LUADs **(C)** and low-risk LUADs **(D)**.

To further gain insights into the biological differences between the high- and low-risk groups, we performed differentially expressed gene analysis and identified 643 DEGs between the two groups. GO enrichment analysis results indicated that these DEGs are associated with microtubule-based movement, humoral immune response, cilium movement, and other biological processes (Figure 4A). KEGG pathway analysis showed that the DEGs are involved in systemic lupus erythematosus and neutrophil extracellular trap formation (Figure 4B). Furthermore, GSEA that incorporates transcriptome data was carried out. Our analysis showed that nucleosome assembly, DNA packaging complex, nucleosome, protein DNA complex, and structural constituent of chromatin are the top five significantly enriched terms in the high-risk group, while in the low-risk group, B cell receptor signaling pathway, complement activation, immunoglobulin complex, T cell receptor complex, and immunoglobulin receptor binding are the top five significantly enriched cellular processes (Figures 4C, D).

**FIGURE 5:**
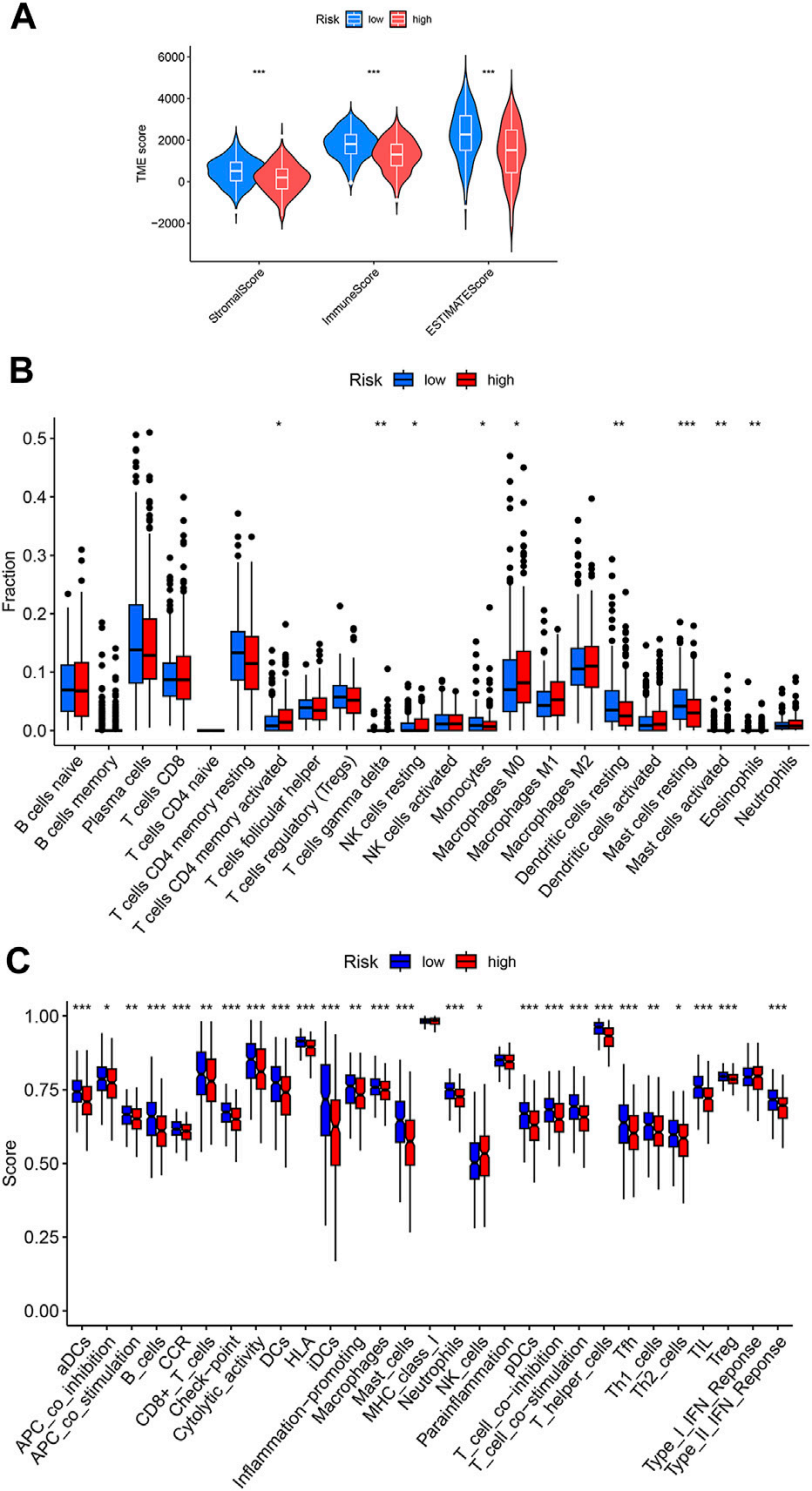
Different immune cell infiltration levels in the high- and low-risk LUADs. **(A)** Violin plots showing the differences of stromal and immune scores between the high-risk and low-risk groups. **(B)** Differences in infiltration degrees of 22 immune cell types in the tumor microenvironment of high- and low-risk LUADs. **(C)** Differences in diverse immune functions activities in the tumor microenvironment of high- and low-risk LUADs. *, *p* < 0.05; **, *p* < 0.01; ***, *p* < 0.001.

Tumor immune microenvironment plays key roles in determining tumor progression. Considering the above GSEA result that showed enrichment of immune related processes in the low-risk group, we speculated that the tumor immune microenvironment is different between the high- and low-risk LUAD groups. According to the ESTIMATE algorithm, the immune scores are significantly lower in the high-risk group than in the low-risk group (Figure 5A), indicating less infiltration of immune cells in high-risk LUAD. We next used CIBERSORT approach to investigate the abundances of diverse immune cell types in the LUAD tissues. As shown in Figure 5B, high-risk LUADs have less infiltration of monocytes, resting Dendritic cells and resting mast cells, but increased infiltration of M0 macrophages. In addition, we analyzed multiple immune functions between the two LUAD groups. Strikingly, among the 29 kinds of immune functions, 25 showed lower function scores in high-risk LUADs than in low-risk LUADs, such as B cells, CD8^+^ T cells, and cytolytic activity (Figure 5C). Together, these results suggest that high-risk LUADs, as classified by our disulfidptosis-related lncRNA model, may have compromised immune responses in their tumor microenvironment, resulting in tumor progression and worse overall survival.

### Mutational landscape of the two LUAD groups classified by the disulfidptosis-related lncRNA model

**FIGURE 6:**
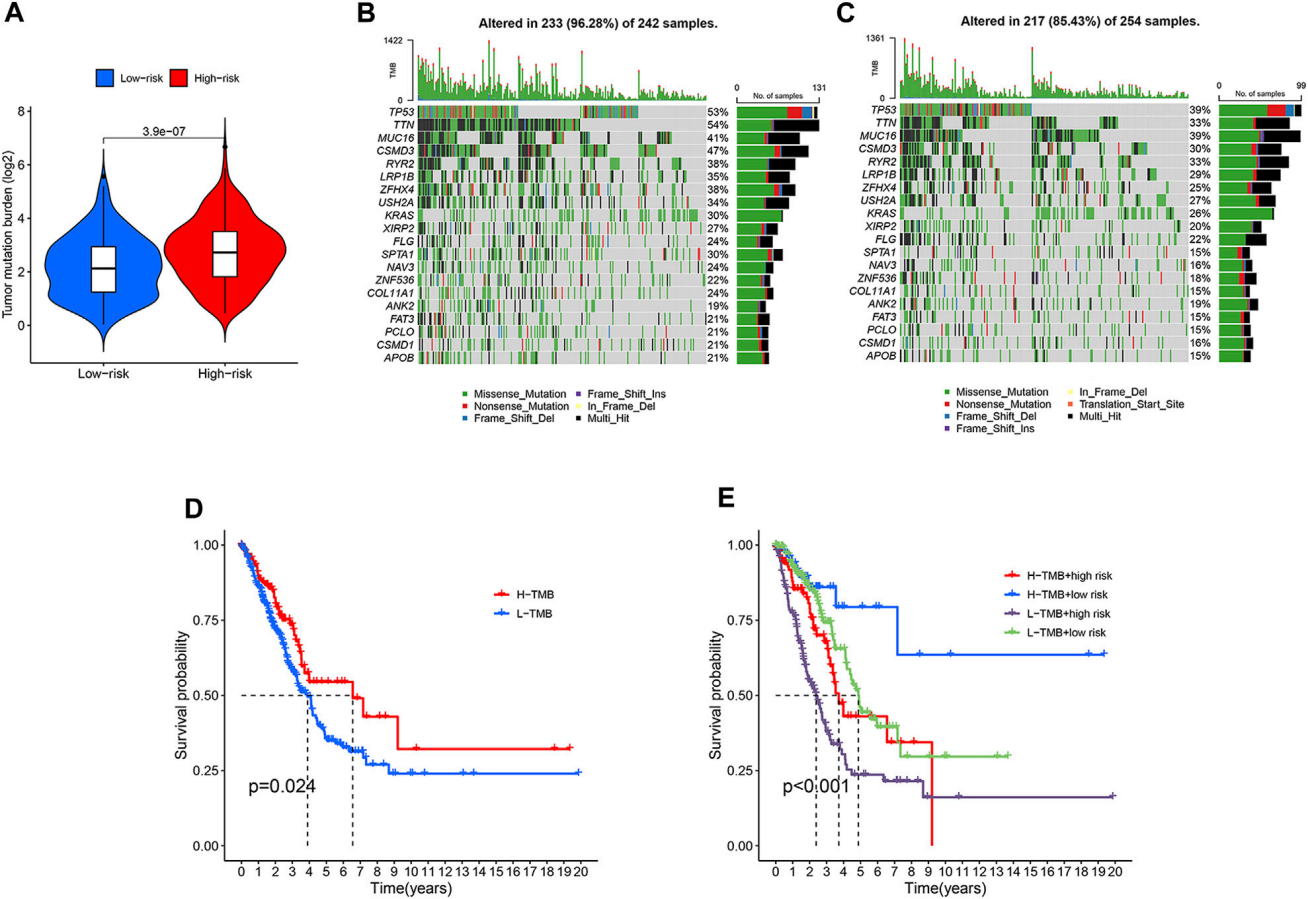
Differential tumor mutational burden and somatic mutation frequencies in the high- and low-risk LUADs. **(A)** Violin plot embedded with box plot showing the difference in the tumor mutational burden between the high-risk and low-risk LUADs. **(B,C)** Mutation frequencies of the top 20 most frequently mutated genes in the high-risk **(B)** and low-risk **(C)** LUADs were shown in the waterfall plots. The upper histograms of the plots represent tumor mutational burden of each sample. The mutation types of each gene are indicated with different colors and the right histograms show the sample number with a certain mutation type of the corresponding genes. **(D)** Kaplan-Meier curves showing the difference in overall survival between LUAD patients with high and low tumor mutational burden. **(E)** Kaplan-Meier curves of overall survival of the four subgroups that are classified based on different tumor mutational burden and different risk score derived from the lncRNA model.

TMB, the number of somatic mutations per megabase of genomic sequence, is a potential predictive biomarker in many solid tumors (Sha et al., 2020). We analyzed and compared gene mutation frequency and TMB between the two LUAD groups. High-risk LUADs exhibited significant higher TMB than low-risk LUADs (Figure 6A). The TMB score of each LUAD sample and the top 20 most frequently mutated genes as well as their mutation types were shown in Figures 6B, 6C. Mutation frequencies of almost all of these genes were higher in high-risk LUADs than in low-risk LUADs. *TP53* and *TTN* were mutated in over half of the high-risk LUADs. High TMB can bring benefits for LUAD patients in their survival, and the overall survival rate is much higher in high TMB patients than in low TMB patients (Figure 6D). We next investigated the effects of risk score and TMB on LUAD patients’ overall survival, patients were divided into four subgroups based on these two factors. We found that patients with high TMB and low risk scores exhibit the best prognosis, their 10-year survival rate is around 60%. In contrast, patients with low TMB and high risk scores have the poorest prognosis, the 5-year survival rate is merely about 25%. There are no significant differences in overall survival between high-risk patients with high TMB and low-risk patients with low TMB, and the survival rate of these patients is between the other two subgroups (Figure 6E).

### Prediction of sensitivity to immunotherapy and other antitumor drugs

**FIGURE 7:**
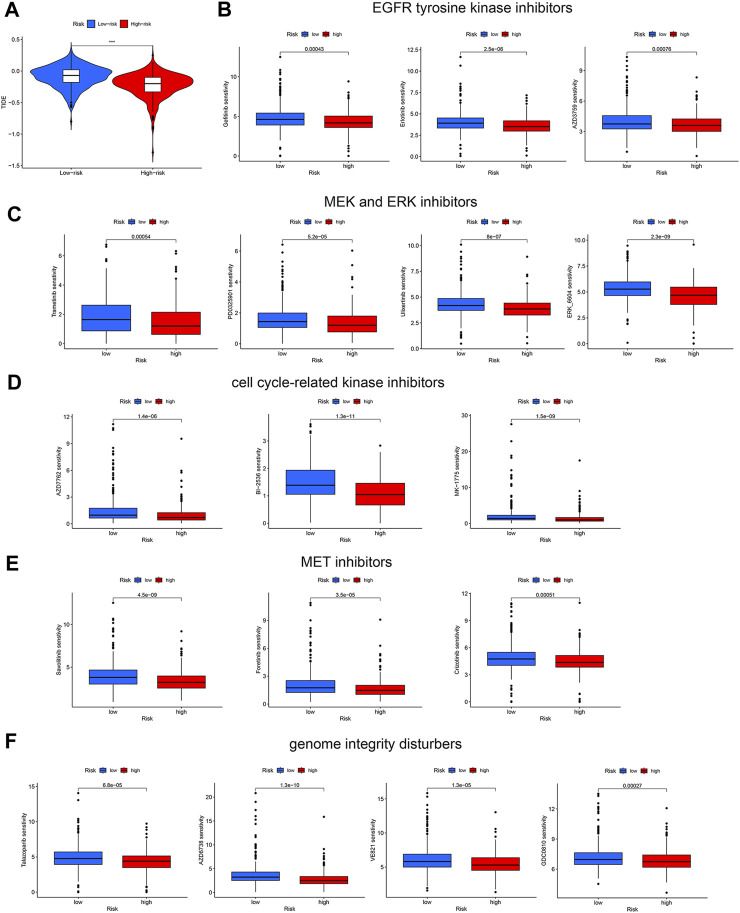
Prediction of response of the two LUAD patient groups to immune checkpoint blockade and other antitumor therapies. **(A)** Violin plots showing the difference in TIDE scores between the high-risk and low-risk LUAD groups. **(B–E)** Different sensitivities of the two LUAD groups to kinase inhibitors, including EGFR tyrosine kinase inhibitors **(B)**, MEK and ERK inhibitors **(C)**, cell cycle-related kinase inhibitors **(D)**, and MET inhibitors **(E)**. **(F)** Different sensitivities of the two LUAD groups to drugs disturbing genome integrity. ***, *p* < 0.001.

Drug resistance is a major cause of cancer relapse and cancer-related death. Immune checkpoint inhibitors (ICBs) have exhibited impressive therapeutic effects in certain cases of NSCLC. To explore the role of our disulfidptosis-related lncRNA model in predicting response to immunotherapy, we analyzed the correlations between LUAD risk score derived from the model and TIDE score. High-risk LUAD patients have significant lower TIDE scores (Figure 7A), suggesting that immune checkpoint inhibitors are more effective in these patients. Since our analysis showed that high-risk LUADs have high TMB (Figure 6A), our results are in line with previous finding that higher TMB was associated with clinical efficacy of anti-PD-1 therapy (Rizvi et al., 2015). In addition, the associations between LUAD risk score and sensitivity to other antitumor agents were also investigated. Drug sensitivity scores were generated by the calcPhenotype function in the oncoPredict package, based on gene expression data of LUAD patients and preprovided training datasets. As compared with patients in the low-risk group, patients in the high-risk group are less sensitive to diverse types of antineoplastic drugs, including EGFR tyrosine kinase inhibitors (gefitinib, erlotinib, and AZD3759) (Figure 7B), MEK and ERK inhibitors (Trametinib, PD0325901, Ulixertinib and ERK_6604) (Figure 7C), inhibitors of cell cycle-related kinases (AZD7762, BI-2536 and MK-1775) (Figure 7D), MET inhibitors (Savolitinib, Foretinib and Crizotinib) (Figure 7E), and drugs that disturb genome integrity (Talazoparib, AZD6738, VE821 and GDC0810) (Figure 7F). These results suggest that our disulfidptosis-related lncRNA model is a potential tool to predict response of LUAD patients to ICBs and other common antineoplastic drugs.

## Discussion

Disulfidptosis is characterized by aberrant disulfide bonding among actin cytoskeleton proteins and subsequent actin network collapse due to cystine overload and NADPH shortage (Liu et al., 2023). Considering cancer cell vulnerability to disulfidptosis, targeting this newly identified cell death form is suggested as a potential therapeutic strategy in cancer treatment. Disulfidptosis-based signature can predict prognosis in various tumor types, including bladder cancer and hepatocellular carcinoma (Chen et al., 2023; Wang et al., 2023). LncRNAs play an important role in regulating malignant behaviors of tumor cells and have been demonstrated as potential biomarkers and targets for cancer diagnosis and treatment (Chi et al., 2019). Hitherto, lncRNAs related to disulfidptosis remain largely unknown and their prognostic significance in LUAD are also unclear. In this study, we identified lncRNAs that exhibit expression correlations with disulfidptosis-related genes, and established a prognostic model for LUAD patients comprised of eight disulfidptosis-related lncRNAs.

Our study identified 127 disulfidptosis-related lncRNAs, and those associated with LUAD patients’ overall survival were screened out for model construction. A risk score model containing eight 8 disulfidptosis-related prognostic lncRNAs was established using LASSO regression analysis. Its predictive efficacy was evaluated in the training and test groups comprising over 500 LUAD patients. A high risk score derived from the model is an indicator for poor overall survival and progression free survival. Tumor stage reflects disease progression and severity. As expected, our analysis showed that tumor stage is an independent prognostic factor for LUAD. Similar to tumor stage, our lncRNA model-derived risk score was proved as a factor with independent prognostic value, and our model is as sensitive as tumor stage to predict three- and 5-year survival. Moreover, one advantage of our model, compared with tumor stage, is that it can distinguish between high- and low-risk patients that are at the same disease stage. Hence, this disulfidptosis-related lncRNA model is an accurate and reliable prognostic predictor for LUAD patients.

Crosstalk between immune cells and tumor cells within the tumor microenvironment have a profound influence on the fate of the later. The quantity and quality of tumor-infiltrating lymphocytes are key factors that forecast prognostic and therapeutic benefits in many types of cancer, such as oral squamous cell carcinoma, HER2-positive breast cancer, and epithelial ovarian cancer (Salgado et al., 2015; Hwang et al., 2019; Shaban et al., 2019; Paijens et al., 2021). In our study, we found that high- and low-risk LUAD patients have different immune activities and immune cell infiltration degrees. High risk scores had significant negative associations with T cell receptor complex, B cell receptor signaling pathway, and immunoglobulin complex. T cell receptors are required for effective antitumor immune responses through participating in tumor antigen recognition and T cell activation (Zhong et al., 2013). B cells mediate humoral immunity and can inhibit tumor growth by secreting immunoglobulins (Wang et al., 2019b). These results indicate a reduced antitumor immune activity in high-risk LUADs. Besides, high-risk LUAD patients had lower immune scores that represent reduced infiltration of immune cells within the tumor microenvironment, according to the ESTIMATE analysis results. Except NK cells that showed a higher function score in the high-risk LUADs, B cells, CD8^+^ T cells, Dendritic cells and macrophages displayed significant lower function scores in these LUADs. Based on these findings, we reason that reduced immune cell infiltration and activity lead to poor prognosis of LUAD patients, and these patients can be distinguished by our disulfidptosis-related lncRNA model.

Despite that immune checkpoint inhibitors demonstrate remarkable survival benefit in NSCLC patients, only a minority of patients respond to them (Dong et al., 2017; Marinelli et al., 2020). Therefore, we wondered whether this disulfidptosis-related lncRNA model can be used as a predictive marker for clinical response to ICBs in LUAD patients. It turned out that high-risk patients had lower TIDE scores, suggesting that these patients are more likely to benefit from immune checkpoint blockade. This may be explained by higher TMB in high-risk patients, as a high TMB is considered as an indicator for better response to immunotherapy (Rizvi et al., 2015; Hellmann et al., 2018). In contrast, we found that high-risk patients are less sensitive to other antitumor therapies, such as EGFR tyrosine kinase inhibitors, MEK/ERK inhibitors, MET inhibitors, and drugs that disturb genome integrity and cell cycle progression.

Somatic driver mutation is a major cause of tumorigenesis and tumor progression. As higher TMB was found in high-risk LUADs, we further investigated genes with high mutation frequencies and compared their differences between the two LUAD groups. Of the 20 most frequently mutated genes, 19 had elevated mutation frequencies in the high-risk group. In the high-risk group there were 10 genes with a mutation frequency greater than or equal to 30%, while in the low-risk group there were only 5. With a mutation frequency of 54% in the high-risk LUADs, *TTN* was the most frequently mutated gene, which may account for the higher TMB in the high-risk group since *TTN* mutations represent high TMB (Oh et al., 2020). Mutation of the tumor suppressor *TP53* gene is among the most common genetic alterations in cancer, which were observed in 53% of patients in the high-risk group. We found higher mutation frequencies of oncogenes such as *MUC16* (Kanwal et al., 2018) and *KRAS* (Tomasini et al., 2016) in high-risk LUADs, which may be another cause, other than reduced immune cell infiltration and activity, of poor prognosis of high-risk LUAD patients.

In conclusion, we identified disulfidptosis-related lncRNAs, based on eight of which we established and validated a prognostic model that can predict independently overall survival of LUAD patients, reflect their immune activity within the tumor microenvironment, and forecast response to immunotherapy, targeted therapy and chemotherapy. This study provides preliminary insights into the association between disulfidptosis and tumor immune response. There are certain limitations in our study. Although the prognostic model has been verified and its accuracy evaluated in over 500 LUAD samples, only TCGA RNA-seq data were used for analysis. Further verification of this model by transcriptome data of other independent LUAD cohorts is needed, and it also remains to be determined whether this model is appliable to data generated by other platforms. In addition, despite that the eight lncRNAs used for model construction show expression correlations with one or more disulfidptosis-related genes, their exact roles in regulating disulfidptosis need further research. Also, the specific molecular mechanisms of disulfidptosis-related lncRNAs in regulating the prognosis of LUAD patients and their response to antitumor therapies remains experimental exploration.

## Data Availability

The datasets presented in this study can be found in online repositories. The names of the repository/repositories and accession number(s) can be found in the article/Supplementary Material.
